# A Highly Effective System for Predicting MHC-II Epitopes With Immunogenicity

**DOI:** 10.3389/fonc.2022.888556

**Published:** 2022-06-16

**Authors:** Shi Xu, Xiaohua Wang, Caiyi Fei

**Affiliations:** Department of AI and Bioinformatics, Nanjing Chengshi BioTech (TheraRNA) Co., Ltd., Nanjing, China

**Keywords:** neoantigen, cancer vaccine, deep learning, IEDB, CD4^+^ T cell, MHC-II

## Abstract

In the past decade, the substantial achievements of therapeutic cancer vaccines have shed a new light on cancer immunotherapy. The major challenge for designing potent therapeutic cancer vaccines is to identify neoantigens capable of inducing sufficient immune responses, especially involving major histocompatibility complex (MHC)-II epitopes. However, most previous studies on T-cell epitopes were focused on either ligand binding or antigen presentation by MHC rather than the immunogenicity of T-cell epitopes. In order to better facilitate a therapeutic vaccine design, in this study, we propose a revolutionary new tool: a convolutional neural network model named FIONA (Flexible Immunogenicity Optimization Neural-network Architecture) trained on IEDB datasets. FIONA could accurately predict the epitopes presented by the given specific MHC-II subtypes, as well as their immunogenicity. By leveraging the human leukocyte antigen allele hierarchical encoding model together with peptide dense embedding fusion encoding, FIONA (with AUC = 0.94) outperforms several other tools in predicting epitopes presented by MHC-II subtypes in head-to-head comparison; moreover, FIONA has unprecedentedly incorporated the capacity to predict the immunogenicity of epitopes with MHC-II subtype specificity. Therefore, we developed a reliable pipeline to effectively predict CD4+ T-cell immune responses against cancer and infectious diseases.

## 1 Introduction

Therapeutic cancer vaccines (1–3) are regarded as the most promising cancer immunotherapies (4–8). The primary therapeutic mechanism of cancer vaccines is to “educate” the immune system to recognize and eliminate tumor cells as foreign substances. From the rationale above, the key of a vaccine design is to identify valuable antigens that can distinguish tumor cells from normal cells. Therefore, previous studies on therapeutic cancer vaccines have involved tumor-associated antigens (TAAs) (9–12) and tumor-specific antigens (TSAs), that is, neoantigens (13–16).

In the past decade, therapeutic cancer vaccines have achieved excellent clinical study results (17–24). For instance, in patients with anti-PD1-refractory/relapsed unresectable Stage III or IV melanoma, BioNTech’s therapeutic cancer vaccine candidate BNT111 combined with cemiplimab elicited durable objective responses (23), which received Food and Drug Administration (FDA) Fast Track Designation in 2021. In another study, treatment with dendritic cell vaccine primed with WT1 mRNA could prevent or delay relapse in 43% of patients with AML in remission after chemotherapy in a Phase II trial (25). These two clinical studies utilized therapeutic vaccines based on TAA.

Therapeutic vaccines based on personalized neoantigens also made remarkable progress. In a clinical trial on melanoma patients conducted by Otto et al., a synthetic long peptide vaccine consisting of multiple epitopes established tumor-specific T-cell responses and demonstrated effectiveness over five years (20, 22, 26). In end-stage colorectal cancer (CRC) patients (3rd line or more advanced), Gritstone’s GRANITE personalized immunotherapy showed a 44% molecular response rate (4/9) by circulating tumor DNA analysis that can be considered as a surrogate endpoint [NCT03639714].

Theoretically, neoantigens are superior to TAA as targets for therapeutic cancer vaccine: although TAAs have relatively higher expression levels in tumor cells, they may still be present in particular types of normal cells at low levels; Her2 and survivin would be good examples (27–30). In contrast, neoantigens originate from mutations and aberrant translations of tumor RNA transcriptome. Consequently, they are “absolutely” specific to tumor cells as normal cells do not have such mutations and aberrant translations. Such “absolute” specificity means that T-cell responses against neoantigens are unlikely to elicit an off-target effect on normal cells. Thus, the safety concern of neoantigen-based personalized vaccines would be minimal.

Despite its theoretical superiority on safety, neoantigen-based personalized vaccines still need to face a technical bottleneck: how to identify T-cell epitopes with sufficient immunogenicity from neoantigens for vaccine design. Especially, MHC-II epitopes are believed to be more necessary than MHC-I epitopes for preventing the immune escape of tumor cells (31, 32, 34). The insufficient capability of predicting MHC-II epitopes has obviously limited the development of neoantigen-based personalized vaccines, resulting in scarcely reported clinical studies involving MHC-II epitopes. Therefore, our research aims to break this bottleneck and provide a powerful tool for developing a neoantigen-based personalized vaccine.

Generally speaking, the conversion of aberrant peptides generated by genomic variations in tumor cells into epitopes eliciting *in vivo* T-cell immune responses is a complex process involving multiple hierarchical levels. Therefore, the prediction and identification of T-cell epitopes should preferably involve multiple levels to reflect complex biological processes. Such a “funnel-like” procedure (35–37) that would eliminate most T-cell epitope candidates would necessarily involve several major steps:

1. Mutation identification
2. Peptide–MHC binding prediction
3. Peptide–MHC presentation prediction
4. Peptide–MHC immunogenicity prediction

Plenty of previous work has been accomplished by various research groups in the relevant field and thereafter generated several well-known software implements:

- The latest version of NetMHCIIpan uses binding and elution datasets deconvoluted by NNalign_MA (38) to predict peptide ligands that can be presented by MHC-I and MHC-II on the cell surface (39, 40).
- MHCflurry improves the pan-allele prediction of MHC-I-presented peptide ligands by incorporating antigen processing and MHC ligandome elution (41).
- ForestMHC applied the deconvolution of polyallelic datasets trained by MixMHCpred based on position weight matrices (PWMs) and MHC-I-presented peptide ligands (42).
- MARIA adopts a multimodal recurrent neural network that summarizes *in vitro* binding measurements, mRNA abundance, and protease cleavage signatures to predict MHC-II-presented peptide ligands (43).

However, the well-known tools listed above never touched the 4th step of the funnel: immunogenicity. Considering the negative selection of T cells during thymus development (44, 45), the vast majority of self-derived peptides will not trigger a downstream immune response even if presented by APC such as DC (46, 47), and such peptides account for 90% of all presented peptides. Obviously, the current antigen presentation prediction tools are NOT the ultimate solutions for the design of neoantigen-based personalized vaccines because even the peptide ligands presented by MHC-I or MHC-II may not be immunogenic at all.

Recently, several emerging studies have taken MHC-I immunogenicity prediction into consideration. For example, deepHLApan incorporated both peptide–MHC complex binding affinity and immunogenicity to predict the T-cell epitope (48). DeepNetBim extracted the attributes of the network as new features from peptide–MHC binding and immunogenic models as a pan-specific MHC-I epitope prediction tool (49).

Nevertheless, there remains an unfilled gap in identifying MHC-II epitopes with sufficient immunogenicity, as neoantigen-driven B-cell and CD4+ T-helper cell collaboration promotes anti-tumor CD8 T-cell responses (50). In this work, we developed an overarching framework to predict MHC-II epitopes: our convolutional neural network (CNN) model predicts the probability of a peptide to be presented to the cell surface by a designated MHC subtype, as well as its immunogenicity to activate immune T cells. The overall research consists of the following parts:

(1) The datasets of peptide presentation and immunogenicity are obtained from an open database (IEDB) (51) and then processed with rigorous organization and cleaning.
(2) We constructed a semiotic-based human leukocyte antigen (HLA)-encoding method with three levels to associate the information of the HLA allele nomenclature, which better represents the characteristics of different MHC subtypes that are not entirely independent or discrete.
(3) The encoded MHC subtypes and peptides are integrated into the deep learning model based on a specially designed CNN.
(4) Independent validation datasets are used to evaluate the model’s prediction performance.

## 2 Materials and Methods

### 2.1 Eluted Ligandome and Immunogenicity Data

The Eluted Ligandome date corresponding to various MHC-II subtypes is downloaded from the IEDB database; T-cell assay data reflecting the immunogenicity of peptides are extracted from the IEDB database. Python scripts are used to resolve raw XML data filtered with the following criteria:

(1) MHC-II alleles include HLA-DP, DQ, and DR β chains, whereas α chains are reasonably omitted as they contribute little to ligand specificity.
(2) Only MHC-II subtypes with explicit 2 fields in the HLA nomenclature such as HLA-DPB1*01:03 are retained.
(3) The peptide length is in the range of 9~25 amino acids, representing 98% of total peptides
(4) Peptide–MHC pairs with controversial assay results are excluded.
(5) MHC-II subtypes with fewer than 10 corresponding peptides are excluded as the data size is too small to train our model, which leads to 65 available MHC-II subtypes.
(6) T-cell assay data are based on wet-lab assays rather than predictions in original dataset’s column named Assay Type.

### 2.2 Negative Elution Training Data Generation

We generate the negative datasets corresponding to elution data treated as positive data from the global maximum dissimilarity scoring matrix based on sequence dissimilarity with an additional NetMHCIIpan binding filter:

(1) Full protein length F is extracted according to its accession ID (GenBank ID) given an eluted sequence P.
(2) We use a window with the same length of P to slide on the full-length sequence F to get a list of candidates from which 10 negative sequences with the lowest sequence similarity compared to the entire positive dataset and the lowest possibility to be eluted sequences calculated by NetMHCIIpan 4.0 as a filter.

In total, we obtained 273,102 non-redundant eluted ligands (as positive data) and corresponding to 61 MHC-II subtypes (**Supplementary Table 1**), amino acids frequency of most prevalence length of top 5 most corresponding restricted peptides of MHC-II subtypes is shown in **Supplementary pdf**; 16,384 (10,131 positive and 6,253 negative) non-redundant T-cell assay data corresponding to 53 MHC-II subtypes (**Supplementary Table 2**).

### 2.3 MHC-II Subtype Encoding Based on Hierarchical Relationship

Antigen presentation and immunogenicity are both closely associated with MHC-II subtypes because peptide ligands are finally presented on the cell surface by MHC-II to T-cell receptors. In order to develop useful tools to predict MHC-II epitopes, we need to “teach” computer programs how to distinguish various MHC-II subtypes. Therefore, setting a reasonable coding method for MHC-II subtypes is an inevitable question. In quite a number of earlier studies, MHC-II subtypes are converted into orthogonal vectors using one-hot encoding. Although a one-hot coding approach is feasible and straightforward, it apparently does not fully reflect biological mechanisms. One-hot coding treats each MHC-II subtype as a unique dimension: for example, in the perspective of one-hot coding, HLA-DRB1*01:01 and HLA-DRB1*01:09 are assumed to have no relation at all, neither are their corresponding ligandomes. However, such an assumption conflicts with real-world biological mechanisms: the evolution of various MHC subtypes can be reflected in phylogenetic trees, and some MHC-II supertypes consisting of multiple subtypes have been characterized by a partially shared ligandome in previous studies.

As an imperfect approach, one-hot coding for MHC-II subtypes may waste lots of training data as it does not recognize the overlapping ligands of closely related MHC-II subtypes. Moreover, one-hot coding would cause the MHC-II subtypes without abundant training data (e.g., fewer than 10 corresponding peptides) to be neglected, as the segregated data amount may not be sufficient for training the model. In order to develop more powerful tools for predicting MHC-II epitopes, we propose a novel coding system that could quantitatively reflect the relation among various MHC-II subtypes. Our goal is to use training data in a more scientific way with maximal utilization and also enable epitope prediction for the MHC-II subtypes without many available data.

**Figure 1:**
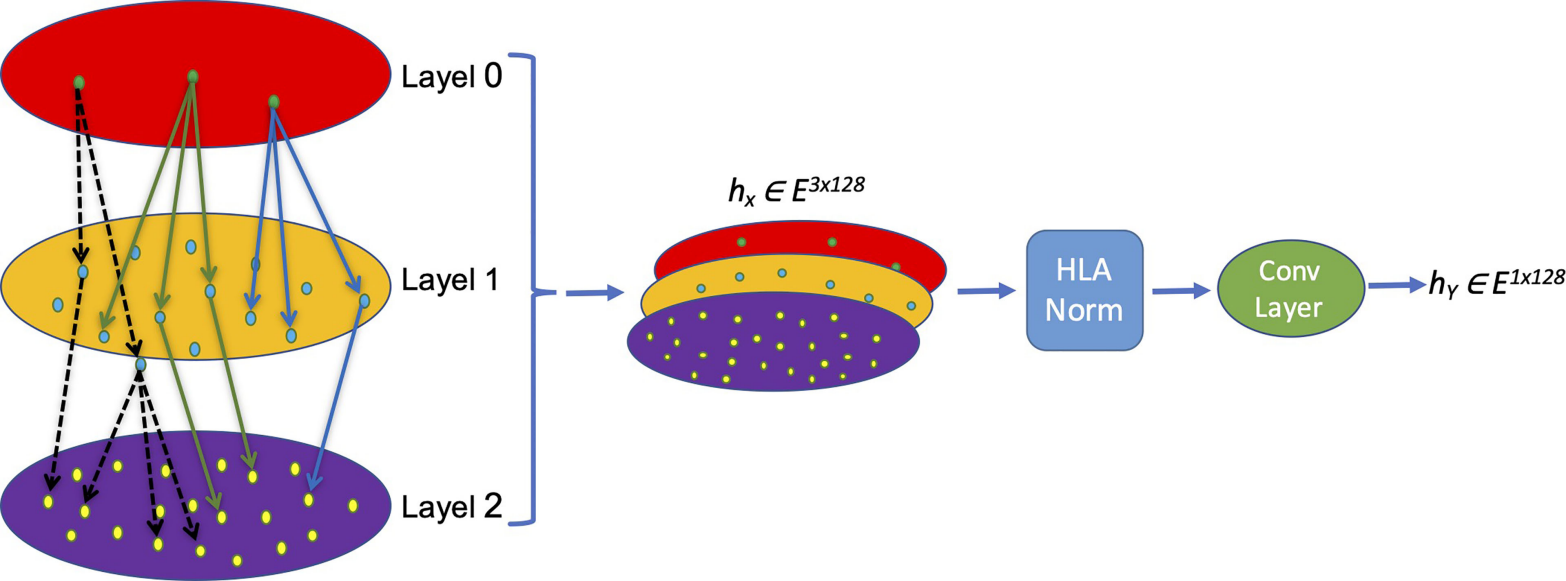
Schema of the MHC-II subtype hierarchical relationship encoding. Each layer representing one field of HLA name is converted into a [1×128] vector and concatenated into a [3×128] matrix, which is normalized and convoluted to get a [1×128] vector for later calculation.


The nomenclature rationale of each HLA allele is like a leaf node based on a tree, which enriches the hierarchical information and truly reflects the categories and associations of different HLA alleles. We creatively propose a new HLA coding method named hierarchical relationship–based HLA encoding, as shown in **Figure 1**. In this model, we regard the HLA gene (HLA-DRB1, DPB1 and DQB1) as layer 0, the first field number (e.g., ‘01’ of DRB1*01) as layer 1, and the second field number (e.g., ‘02’ of DRB1*01:02) as layer 2. We encode each single layer according to an [99×128] embedding table to get an *E ∈ R*^1×128^ vector that represents each layer so that on the single layer, the same symbols have the same biological means while different symbols are discrete and orthogonal to each other mathematically. Afterwards, a transition matrix is adopted to transform the concatenated three-layer encoding matrix [3×128] into a one-dimensional vector [1×128] for later model training.

$$embedding=concat\left(embedding_{0}:embedding_{1}:embedding_{2}\right)$$

**Equation 1.** *embedding*_0_, *embedding*_1_, *embedding*_2_, represents the coding information of each layer, respectively; in each layer, the coding container size is [99×128].


$$e\;=\;\sigma (\sum_{i=0}^{3}E_{i}W_{i})$$

**Equation 2.** Convolution of feature extraction, *W_i_* is the transfer matrix representing the weight of each layer. *E_i_* is the information coding of each layer; ***e*** is the integrated HLA embedding value.

### 2.4 Normalization of HLA Embedding Value

The obtained HLA embedding value needs to be normalized before feeding to the deep learning model. Batch normalization (BN), a commonly used method, is used to normalize the whole batch of the dataset to a standard Gaussian distribution (52) so that differences in distinct data distribution from different samples can be normalized according to Equation 3:


$$BN\left(X\right)\;=\;\frac{\left(x-\upsilon \right)}{\sqrt[{2}]{\sigma^{2}+\;u}}$$

**Equation 3.** Batch normalization. *v* and *σ*^2^ are the per-dimension mean and variance, respectively. Arbitrarily, the constant *u* is added in the denominator for numerical stability.

On the contrary, layer normalization (LN) normalizes all features of each sample in the sample scale (53) according to Equation4:


$$LN\left(x\right)\;=\;\frac{\left(x-\upsilon^{l}\right)}{\sqrt[{2}]{\sigma^{2l}+u}}$$

**Equation 4.** Layer normalization *v* and *σ*^2^ are the per-dimension mean and variance, respectively. Arbitrarily, constant *u* is added in the denominator for numerical stability for each single layer *l*.

Both batch normalization and layer normalization could be used to avoid gradient disappearance or gradient explosion caused by excessive fluctuation of the input value, so as to simplify subsequent model training. However, they still have substantial differences: batch normalization depends more on the statistical parameters between different samples; thus, feature extraction and normalization calculation within a single sample are insufficient, whereas layer normalization eliminates the characteristic relationship between different samples in a batch and only normalizes different eigenvalues in the same sample. Because of the reasons described above, both methods are not very suitable for current HLA embedding normalization. Because we need to consider not only the characteristics of the same layer but also the impact of differences at different layers, we developed a new method of HLA normalization (HLAN):


$$\mu_{c}^{l}\;=\;\frac{1}{CH}\sum_{1}^{c=3}\sum_{1}^{H}x_{ci}^{l}$$

$$\sigma_{l}^{l}\;=\;\sqrt{\frac{1}{CH}\sum_{1}^{3}\sum_{1}^{H}{\left(x_{ci}^{l}-\mu_{c}^{l}\right)}^{2}}$$

$$HLAN\left(x\right)\;=\;\frac{\left(x_{ci}^{l}-\;\mu_{c}^{l}\right)}{\sqrt[{2}]{\sigma_{c}^{l}\;+\;}\epsilon }\times \alpha +\beta$$

**Equation 5.** HLA normalization equation ***µ*** is the mean value based on different levels, *σ* is the level variance, *x* is the input value *C* is the layer according to the HLA-named system, and *H* is the length of the input value.

After normalization, the features are integrated through a convolution layer, and the final output results that are used as the input of the subsequent deep learning model are as follows:


$$h_{x}=conv\left(HLAN\left(x\right)\right)$$

**Equation 6.** Convolution layer to integrate an HLAN result.

### 2.5 HLA-Encoding Fusion Layer

**Figure 2:**
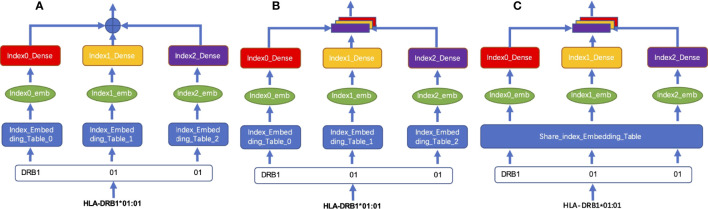
Schema of HLA-encoding fusion layer. **(A)** integrates sequence information by means of direct addition; **(B)** integrates the sequence information by means of concatenation; **(C)** shows that the sequence information is integrated by concatenation after the weight is processed by using the shared index embedding table.

We tested two different coding fusion layer schemas to fuse hierarchical representations from different layers representing an MHC-II subtype nomenclature in **Figures 2A, B**. Considering that the numbers representing MHC-II subtypes are sparse in some datasets, inadequate training may occur during model training, we merged the embedding table at each level as shown in **Figure 2C**; shared parameters are calculated as the same embedding table called HLA_Norm.

### 2.6 Variable Length Peptide Encoding

We first built a 21-character vocab, which uses J as the initial letter for the completion of the lengths of peptides, which are less than 25, plus 20 single-letter symbols of amino acids:

vocab = [“J”,“A”,“C”,“D”,“E”,“F”,“G”,“H”,“I”,“K”,“L”,“M”,“N”,“P”,“Q”,“R”,“S”,“T”,“V”,“W”,“Y”]

A [21×128] size embedding table based on random normal distribution is developed according to the vocab shown in **Supplementary Table 5**.

For each input peptide sequence, we completed its length to 25 with the letter “J” and then converted each letter into a [1×128] vector according to its position in vocab to tokenize the whole sequence and finally get a [25×128] matrix presenting the input peptide.

### 2.7 HLA Subtype and Peptide Sequence Fusion Encoding

**Figure 3:**
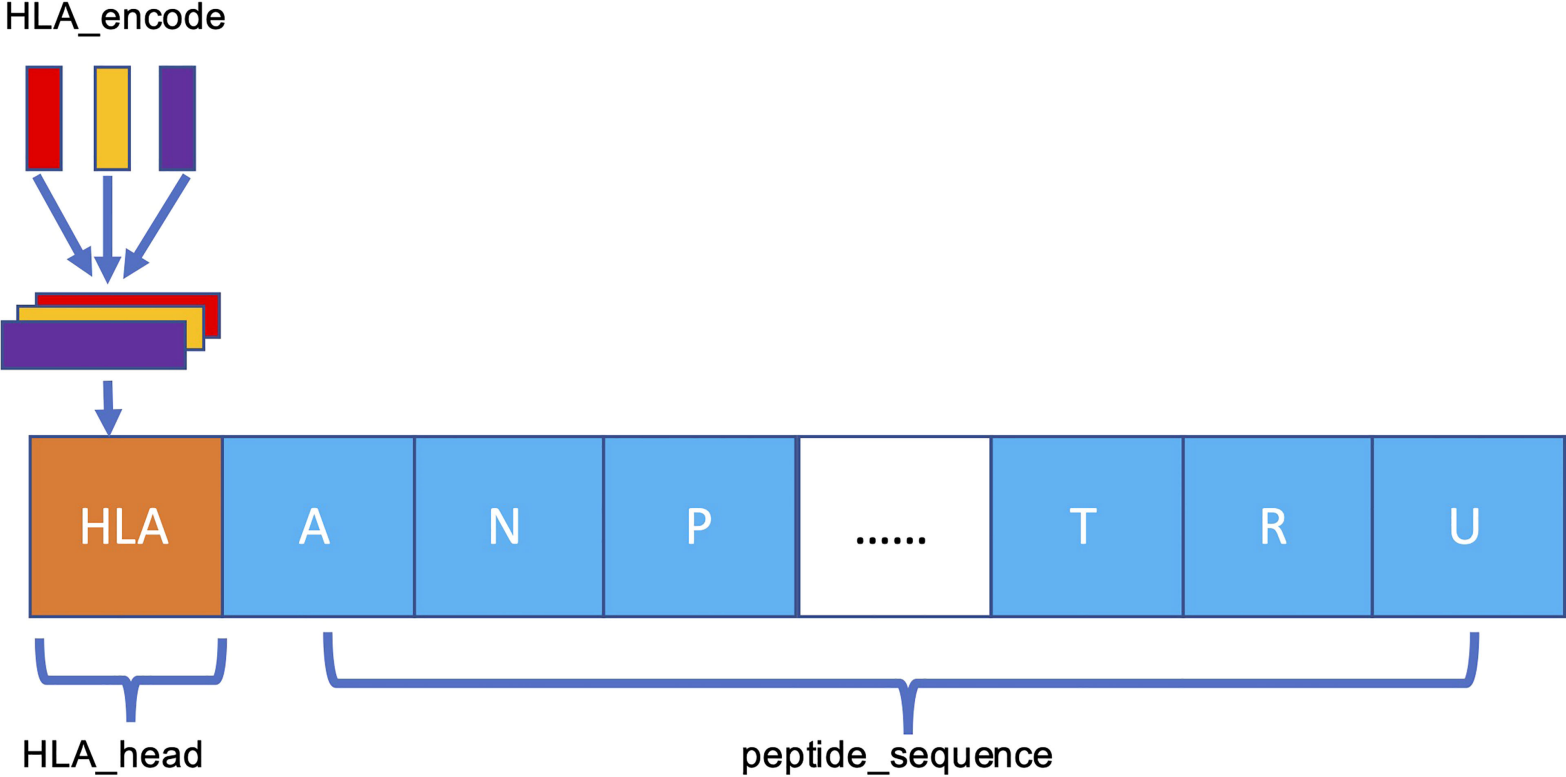
Schema of HLA–peptide fusion encoding. The orange HLA_head is the result generated in **Figure 2C**. HLA_head is loaded on top of peptide sequence in concatenation mode to form a new constituent sequence containing HLA_head and peptide.

The MHC-II subtype and peptide sequence are paired, concatenated (**Figure 3**), and sent to our model for further training and testing.

To characterize an HLA-peptide sequence after pairing, we need a unified model that can extract the paired information. Compared with the recurrent neural network, a full CNN can better model the information of adjacent positions. A one-dimensional CNN can be expressed as follows:


$$p\left(x\right)\;=\;f*X\;=\sum_{1}^{N}f^{c}*x^{s}$$

**Equation 7.** *f* is the convolution kernel, * is the convolution operator, and *X* is the input value. *f^c^* is a one-dimensional convolution kernel of ***c*** dimension, and *x^s^* is the input value decomposed according to its own dimension.

### 2.8 10-Fold Cross-Validation

Ten-fold cross-validation is applied to evaluate model robustness. Before training, the dataset is randomly partitioned into 10 non-overlapping subsets. The cross-validation process is repeated 10 times, with each subset used as a validation set while the remaining subsets are utilized as the training set. The results of the cross-validation sets are averaged to obtain the final result. One hundred epochs are executed, and the model is saved if the validation accuracy is better than previous epochs.

## 3 Results

### 3.1 The Architecture of FIONA

**Figure 4:**
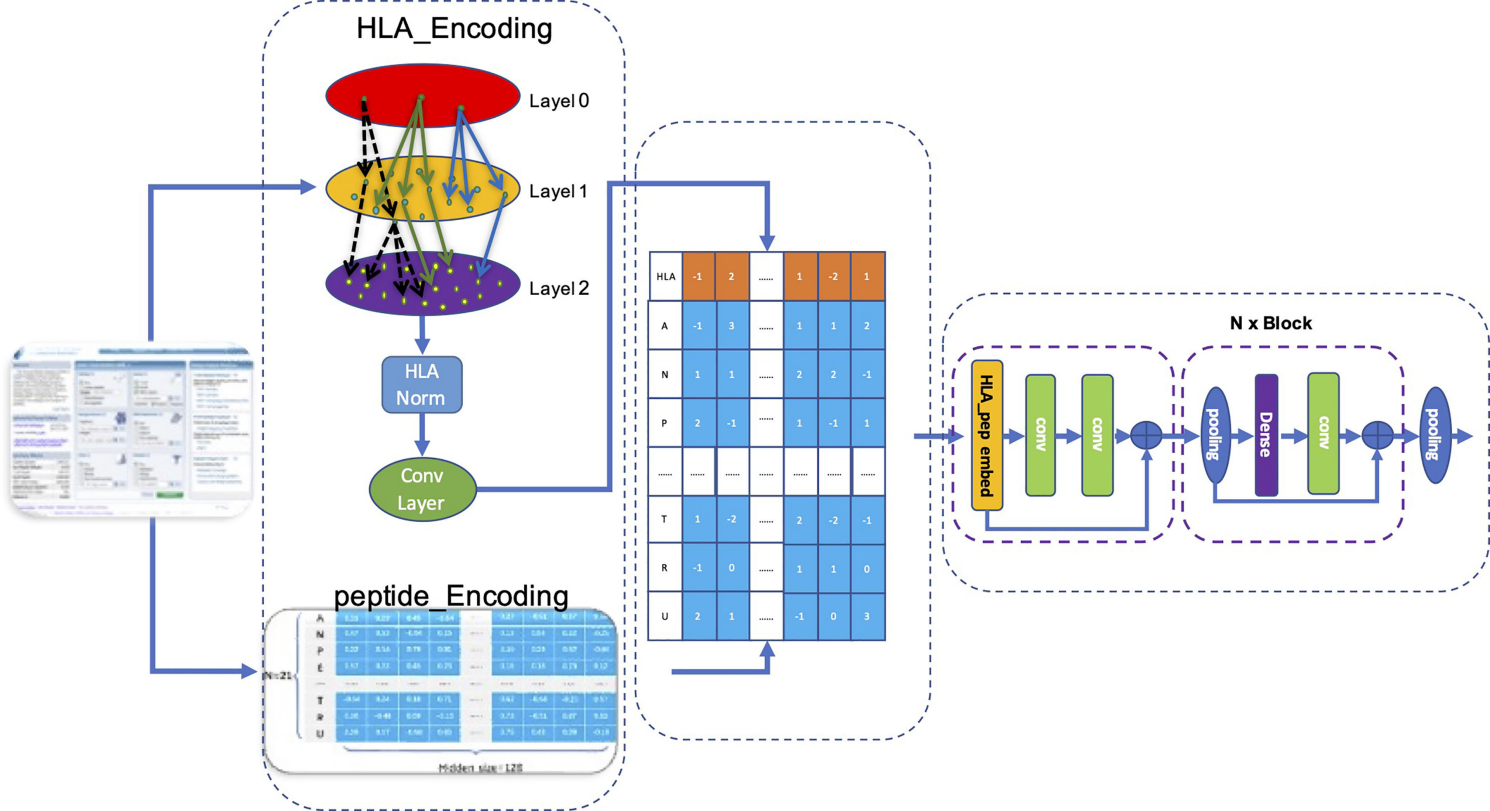
Architecture of FIONA. The dataset is downloaded from the IEDB database according to Section 2.1. The first dotted box contains an HLA_head and peptide, which are encoded separately and then integrated for feature transformation. The middle part contains an HLA_head, and peptide_Embedding represents sequence feature information. The right dotted box is our CNN model for training and prediction.

We used the matrix *p(x)* obtained by matrix transformation in Equation 6 that converts a one-dimensional vector sequence of the MHC-II subtype and peptide into a [26×128] matrix as input for the model to predict whether a peptide will be presented to the cell surface (FIONA-P) or trigger immunogenicity (FIONA-I) given a specific MHC subtype. In order to implement the above 2 predictive functions, we constructed two models with different training datasets (presentation and immunogenicity) explained in Section 2.1 and Section 2.2 with the same architecture shown in **Figure 4**. FIONA includes a CNN layer for prediction, which focuses on integrating and extracting overall features from MHC-II subtype–peptide pairs. In this process, HLA embedding and peptide embedding are integrated to play a synergistic role in improving the prediction performance. Additionally, in order to improve the prediction ability of our model, we added multiple pooling layers in the convolution layer to extract and integrate features.

Our model accepts fused HLA_Peptide embedding as input. Referring to Resnet’s design pattern shown in the left of **Figure 4**, we created several aggregation modules in the form of blocks for stacked layers, connected layers, and convolution layers successively named BlockConv. The final convolution layer aggregates the internal characteristics of each embedding into a vector.

### 3.2 Ablation Experiment

We conducted ablation experiments to validate our HLA-encoding schema and its impact on the overall results by eliminating the HLA_Norm layer or replacing the normalization layer with batch normalization and layer normalization individually. We divided the comparison into two parts: the first part is based on HLA_ Embedding using different encoding and normalization methods, and the second part partially modifies the architecture of our model to find out the impacts of these modifications on the performance of our model.

As shown in **Table 1**, the ablation test shows that our MHC subtype hierarchical relationship–encoding method greatly outperforms the traditional one-hot method regardless of subsequent normalization methods on both presentation data and immunogenicity data. In addition, the HLA_Norm method has the best performance on both presentation and immunogenicity datasets compared to the Batch Norm and Layer Norm. Meanwhile, the final architecture consisting of con_ANA and BlockConv has the best performance among all tests including eliminating the HLA coding content, which leads to a dramatic decrease of ROC and PR values.

**Table 1:** Results of the ablation experiment. MSE (mean-squared error), AUC (area under the curve), and PR (precision rate) are evaluation indicators.

Method	MHC-II presentation			MHC-II immunogenicity		
	MSE (test)	AUC (test)	PR (test)	MSE (test)	AUC (test)	PR (test)
PE+HLA_Norm+con_ANA+BLOCKConv	**0.0421**	**0.9391**	**0.9513**	**0.1819**	**0.8876**	**0.9344**
PE+Batch_Norm+con_ANA+BLOCKConv	0.0534	0.9242	0.9442	0.2049	0.8433	0.8839
PE+Layer_Norm+con_ANA+BLOCKConv	0.0610	0.9197	0.9328	0.2031	0.8340	0.8581
PE+HLA_Norm+add_ANA+BLOCKConv	0.0781	0.9038	0.9291	0.2274	0.8014	0.8230
PE+HLA_onehot+con_ANA+BLOCKConv	0.1042	0.8467	0.8835	0.2625	0.7637	0.7784
PE+BLOCKConv	0.2427	0.8046	0.8476	0.4691	0.5745	0.5872
PE+HLA_Norm+con_ANA+Conv	0.0578	0.8656	0.9103	0.2128	0.7877	0.8237

PE refers to the general peptide embedding, Batch_Norm, Layer_Norm, and HLA_Norm refer to the different HLA normalization methods described in section 2.4, while con_ANA is used to refer to the concatenate peptide_Embedding and HLA_Embedding header to get a [26×128] matrix for the following step calculation, add_ANA refers to peptide_Embedding, and HLA_Embedding is processed by direct addition, which is mentioned in Section 2.5.

Bold means highlight superiority of our model.

### 3.3 FIONA-P Favors Balanced Positive and Negative MHC-II Peptide Presentation Data

**Figure 5:**
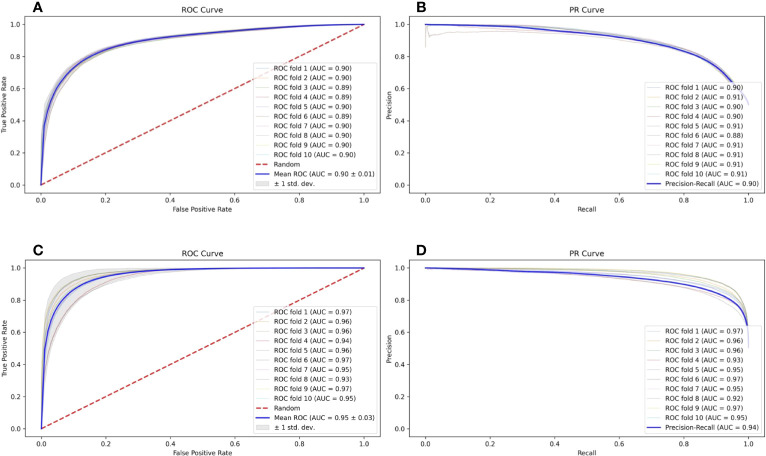
Influence of balanced and unbalanced data ratios on FIONA-P. **(A, B)** are the ROC (receiver operating characteristic) curve and PR (precision and recall) curve of unbalanced data (AUC=0.90, PR=0.90), respectively, while **(C, D)** are the ROC curve and PR curve of balanced data (AUC=0.94, PR=0.95), respectively.

In a natural environment, the proportion of presented antigens compared to non-presented peptides degraded by protease is relatively low; therefore, the unbalanced data amount should theoretically and more faithfully reflect the actual situation. However, the unbalanced data amount of positive and negative samples is a great challenge to the construction and optimization of the deep learning model. Here, we selected a specific number of samples from multiple negative samples generated by the method mentioned in Section 2.2 to build 2 datasets with relatively balanced and unbalanced positive and negative ratios (positive data to negative data = 1:1 and 1:5, respectively) to compare the influence to our model FIONA-P. As shown in **Figure 5**, FIONA-P has a better performance for balanced datasets, especially in terms of the performance of PR, which has a pronounced degradation if unbalanced data are used.

Similarly, we also compared the performance of natively uneven immunogenicity data from the IEDB and artificial synthetic datasets after randomly reducing a portion of the positive data (positive data to negative data = 1.62:1 and 1:1, respectively) to test the performance of the FIONA-I model under such circumstances. The test results show only minor changes in terms of ROC and PR.

### 3.4 FIONA-P Achieves Comparable Performance

**Figure 6:**
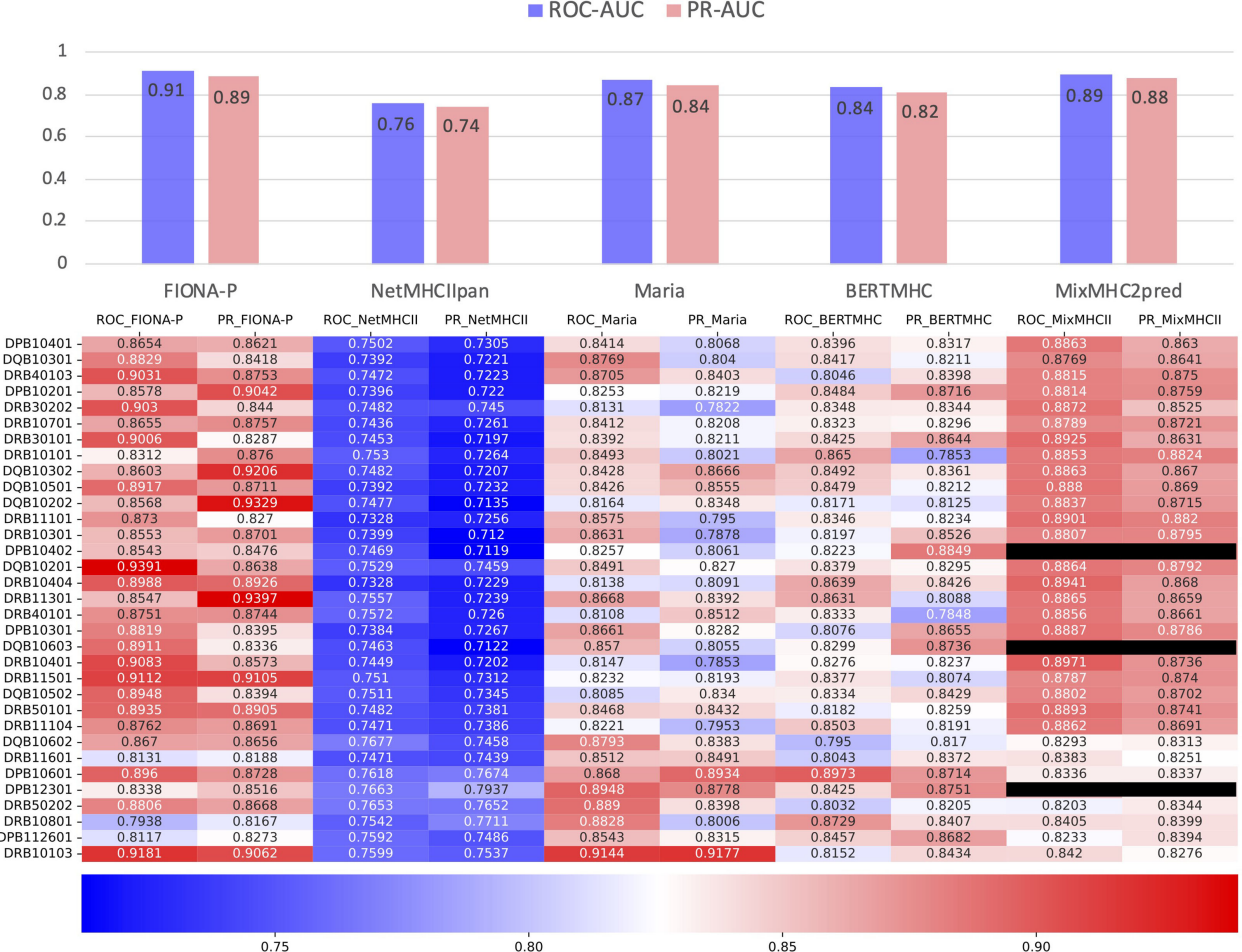
Comparison of FIONA-P and other prediction tools on the presentation data of all available MHC-II subtypes. The black ones indicate that those MHC-II subtypes are not supported.

The IEDB benchmark dataset is often used to compare the performance of different binding prediction tools. However, these datasets are usually intracellular binding data rather than elution data. To test the ability of the presentation prediction of several existing MHC-II epitope tools [Maria, NetMHCIIpan4.0, BERTMHC (54), and MixMHC2pred (55)], we used an independent dataset from the University of Tübingen (56) that contains 142,625 naturally eluted ligands from 29 tissues across 42 MHC-II subtypes (33 MHC-II subtypes in total after omitting the α chains of MHC, **Supplementary Table 3**). The independent dataset is deduplicated by sequence and the corresponding MHC-II subtypes compared with the training dataset. All the supported MHC-II subtypes that overlap the MHC-II subtypes of the independent dataset are tested. For all tools, our FIONA-P model achieved the best performance for 25 out of the 33 MHC-II subtypes, especially in subtypes with higher corresponding eluted peptides as shown in **Figure 6**. Our model has shown a bit of advancement compared with MixMHC2 and great improvement compared with other tools. However, MixMHC2 only supports 38 MHC-II subtypes; thus, 3 of unsupported MHC-II subtypes have no available results in this comparison. Our model not only supports 65 MHC-II subtypes by direct training but is also able to predict the peptide presentation of corresponding untrained MHC-II subtypes by our new breakthrough HLA hierarchical encoding method. Since the number of supported MHC-II subtypes is also very important in epitope prediction, our model has greatly broadened the scope of available MHC-II subtypes.

### 3.5 FIONA-I Improves Positive Prediction Value of True Neoantigen Through Validation of Curated Neoantigen Dataset

As previously discussed, only a small proportion of peptides presented by APC can trigger the downstream immunogenicity of T cells, resulting in a fairly low false-positive rate (FPR), which is presumably one of the main reasons that cancer vaccines do not have enough clinical benefits since these vaccines cannot load sufficient epitopes to inhibit the immune escape of cancer cells given such high FPR.

We used a fully manually annotated neoantigen database, NEPdb (57), newly published in 2021 to demonstrate that our FIONA-I model substantially improves the positive predictive value (PPV) of neoantigen prediction compared to the antigen presentation model. All MHC-II neoantigen data entries containing DP, DQ, and DR alleles were retrieved from the NEPdb, which contains 182 positive and 3,508 negative epitopes across 31 different MHC-II subtypes (**Supplementary Table 4**). FIONA-I, FIONA-P, and other MHC-II epitope tools (Maria, NetMHCIIpan4.0, BERTMHC, and MixMHC2pred) are used to calculate the PPV with a default parameter setting. Maria/BERTMHC directly returns ‘0’ for negative and ‘1’ for positive, NetMHCIIpan4.0 and MixMHC2pred take top 10% peptides as positive; all the MHC-II subtypes that are not supported by these tools are neglected. As shown in **Table 2**, FIONA-I raises the PPV from 22.51% (mean PPV of FIONA-P, Maria, NetMHCIIpan4.0, BERTMHC, and MixMHC2pred) to 40.27%, obtaining a near doubling of the increasement. The results showed that FIONA-I could improve the PPV significantly and retain the sensitivity at 0.89, indicating that the immunogenicity model could greatly contribute to high-confidence neoantigen identification.

**Table 2:** Results of immunogenicity prediction of MHC-II-restricted epitopes in terms of sensitivity, specificity, and positive predictive value (PPV).

Tools	MHC-II Immunogenicity		
	PPV	Sensitivity	Specificity
FIONA-I	**0.4027**	**0.8846**	**0.9319**
FIONA-P	0.2188	0.7340	0.8640
NetMHCIIpan 4.0	0.1295	0.9271	0.6767
BERTMHC	0.1683	0.8093	0.7925
Maria	0.3279	0.7846	0.9166
MixMHC2pred	0.2812	0.7425	0.9032

Bold means highlight superiority of our model.

### 3.6 Web Service

We developed a user-friendly web interface (http://therarna.cn/fiona.html), allowing visitors to quickly query whether peptides would be presented or able to trigger an immune reaction given the specific MHC-II subtypes.

## 4 Discussion

Since 2018, a couple of useful tools for antigen presentation prediction have been reported. For example, Gritstone has published its proprietary software for predicting the MHC-I epitopes presented on the cell surface; similarly, MARIA is capable of predicting the MHC-II epitopes presented on the cell surface. Both tools use the same underlying hypothesis: antigen processing by proteases, antigen abundance, and peptide–MHC interaction are 3 important factors that participate in antigen presentation. Therefore, both tools involved these 3 factors into the algorithm training and by far outperformed early versions of NetMHCIIpan and MHCFlurry.

The 3-factor theory of antigen presentation actually makes good scientific sense: short peptides need to be cleaved from long peptides by proteases to enable their binding to MHC-I or MHC-II; antigen abundance measured by the mRNA level determines the amount of MHC ligands displayed on the cell surface to be recognized by a T-cell receptor, whereas peptide–MHC interaction would tell which ligands are more favorable to be displayed by MHC. However, there might be better ways to integrate these 3 factors into antigen presentation prediction. For instance, in the algorithm structure of MARIA, antigen presentation is simplified to a cleavage score, peptide–MHC interaction is simplified to an HLA-DR binding score, and antigen abundance is standardized as mRNA TPM (transcript per million); afterwards, the 3 types of data from different dimensions were put into the algorithm training. We are not saying “that approach is not right,” but we seriously want to discuss what a better model should be. Biologically, long peptide cleavage by proteases occurs before short peptides interact with MHC. Therefore, it is more reasonable to develop a tool to enumerate all short peptides generated from a long peptide by protease cleavage, and the pool of short peptides would be the input of the next-step antigen presentation prediction. Moreover, in the antigen presentation process, antigen abundance would no longer be a limiting factor once it exceeds a reasonable level, which has been proven in the neoantigen meta-analysis of TESLA (58). Therefore, we may use TPM>35 proposed by TESLA as a cut-off point of antigen abundance. In other words, the antigens whose expression levels are above the cut-off point should be regarded as “abundant” to be presented. Furthermore, in a natural infection caused by an exogenous virus or bacteria, all pathogen-related antigens should be regarded as “abundant,” even though their expression levels could hardly be standardized as TPM.

Based on the mechanistic analysis above, antigen processing had better been analyzed with a separate upstream tool, whereas antigen abundance could be reasonably simplified to a criterion of TPM >35. Therefore, our antigen presentation prediction tool focuses more on peptide–MHC interaction. We would not recommend MARIA’s approach of oversimplifying the peptide–MHC interaction to a binding score because the amino sequences of peptide ligands as well as MHC complex may reveal important information relevant to the antigen presentation process. For example, previous studies confirmed that peptide-MHC binding affinity reflected as IC50 (nM) does not accurately reflect the stability of the peptide–MHC complex. Thus, the sequences of peptide ligands would provide information in more than one dimension. Taking all the foresaid into account, our antigen presentation prediction tool involves the sequences of peptide ligands and MHC into deep learning and therefore avoids the issue of oversimplification. This could be a possible explanation that our model outperforms the well-known tools.

Compared to antigen presentation, predicting the immunogenicity of MHC ligands is more challenging due to the lack of powerful theories. As previously discussed, there is a scientifically sound 3-factor theory that explains the mechanism of antigen presentation, and this theory effectively guided the development of multiple prediction tools. In contrast, the root cause of immunogenicity is more difficult to interpret.

Immunogenicity is shaped by a T-cell-negative selection; thus, the real challenge of immunogenicity prediction is the limited understanding of the mechanism of a T-cell-negative selection. A T-cell-negative selection process in thymus removes T cells reactive to self-antigens from the T-cell repertoire and therefore provides protection against unwanted T-cell responses. A T-cell-negative selection determines which MHC ligands will NOT elicit an immune response, whereas other MHC ligands may still encounter the corresponding TCR in the T-cell repertoire.

So far, the T-cell-negative selection process is still a “black-box,” and there is no powerful theory that clearly interprets its delicate mechanism. Especially, no theory could define what factors constitute the “sufficient condition” to trigger a T-cell-negative selection. At least, self-antigen alone does not constitute the “sufficient condition.” A T-cell-negative selection does NOT remove all T cells that recognize the MHC ligands derived from self-antigens, and such complexity is endorsed by 2 facts in clinical studies:

1. Self-reactive T cells are present in patients with autoimmune diseases (59).
2. A peptide vaccine could elicit T-cell responses against TAA in cancer patients (2).

The lack of a robust theory to interpret a T-cell-negative selection makes it challenging to predict immunogenicity. All software tools for predicting immunogenicity, including ours, are based on an empirical approach: the tools are trained with T-cell assay data that distinguish immunogenic peptides from non-immunogenic ones, matched with MHC subtypes. Of course, even an empirical approach could solve many problems. For example, our trained software could achieve PPV at 40.27% on an independent dataset. Nevertheless, the limitation of the empirical approach should not be forgotten: such methodology requires tremendous T-cell assay data to train a functional model. For those MHC subtypes that do not have many corresponding T-cell assay results, the empirical approach cannot be used. Based on our discussion above, a more accurate immunogenicity prediction tool would rely on the emergence of a more robust theory that interprets the mechanism of the T-cell- negative selection mechanism. By then, it might be possible to deduce the immunogenicity of peptide ligands based on the host’s MHC genotype and proteome information.

Our study proposed a systematic workflow that could identify MHC-II restricted epitopes that can be presented on the cell surface and elicit immune responses. This tool could be of great usefulness for identifying potential epitopes from cancer neoantigens and paving the way of designing effective cancer therapeutic vaccines.

## Data Availability Statement

The original contributions presented in the study are included in the article/**Supplementary Material**. Further inquiries can be directed to the corresponding author.

## Author Contributions

SX is the first author of this article. SX and CF designed the concept and experiments. XW performed the ablation experiments. SX and CF prepared the data for training. CF implemented the negative data generation algorithm. XW implemented the CNN model. CF prepared the IEDB data and plotted the final figures and tables. CF and XW performed statistical analysis. SX and CF wrote the paper with input from XW. All authors contributed to the article and approved the submitted version.

## Conflict of Interest

Authors SX, XW, and CF were employed by the company Nanjing Chengshi BioTech (TheraRNA) Co., Ltd.

## Publisher’s Note

All claims expressed in this article are solely those of the authors and do not necessarily represent those of their affiliated organizations, or those of the publisher, the editors and the reviewers. Any product that may be evaluated in this article, or claim that may be made by its manufacturer, is not guaranteed or endorsed by the publisher.

## Supplementary Material

All the training and validation datasets mentioned in this work including human MHC-II elution and immunogenicity data from IEDB plus individual datasets from Tübingen and NEPdb are **Supplementary Tables 1–4** respectively. Full table of initial table of peptide encoding and WebLogo of sequence motifs of top 10 MHC-II subtypes corresponding peptides is shown in **Supplementary pdf file**.

## Abbreviations

APC, antigen-presenting cell; MHC, major histocompatibility complex; HLA, human leukocyte antigen; CNN, convolutional neural network; CRC, colorectal cancer; TAA, tumor-associated antigens; TSA, tumor-specific antigens; AML, acute myeloid leukemia; DC, dendritic cell; TCR, T-cell receptor; ROC curve, receiver operating characteristic curve; AUC, area under the curve; PR curve, precision and recall curve.
