# Supplementary material for: A Highly Effective System for Predicting MHC-II Epitopes With Immunogenicity
Source: Front Oncol. 2022 Jun 16;12:888556. doi: 10.3389/fonc.2022.888556 (PMC9246415; doi:10.3389/fonc.2022.888556)
Supplement: Supplementary file 1 [file Image_1.pdf]

Supplementary Material

1 Supplementary Figures and Tables

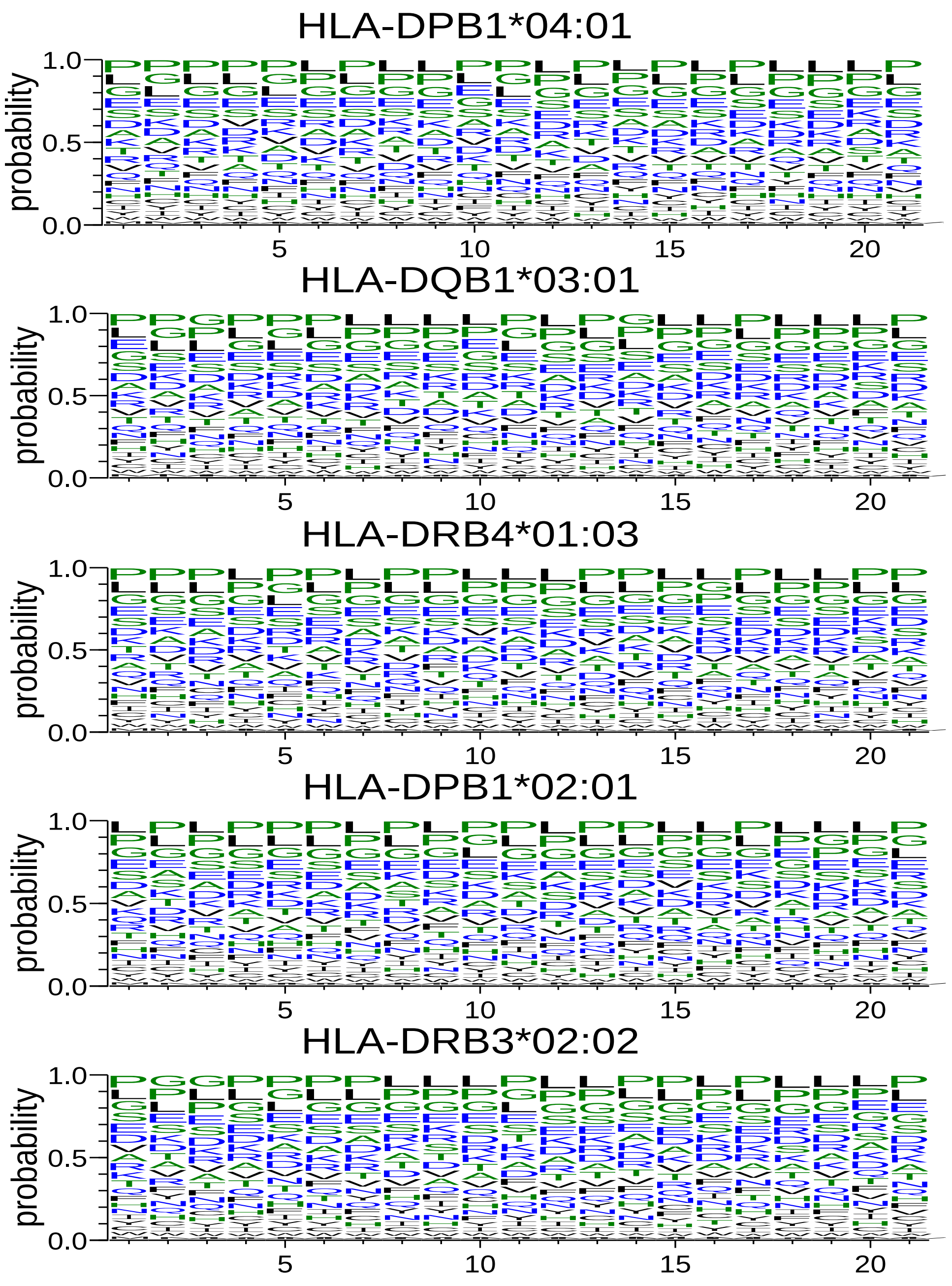

Supplementary Figure 1. Amino acids frequency of most prevalence length of top 5 most corresponding restricted peptides of MHC-II subtypes. Since there is no significant sequence motifs in the ligandome, probability model is used to draw the figures.
